# High Expression of KIF26B in Breast Cancer Associates with Poor Prognosis

**DOI:** 10.1371/journal.pone.0061640

**Published:** 2013-04-09

**Authors:** Qun Wang, Zong-Bin Zhao, Geng Wang, Zhen Hui, Ming-Hua Wang, Jun-Feng Pan, Hong Zheng

**Affiliations:** 1 Department of General Surgery, Taihe Hospital, Hubei, Shiyan, People's Republic of China; 2 Department of Preventive Medicine, Taihe Hospital, Hubei, People's Republic of China; Health Canada, Canada

## Abstract

To date, a great number of studies have demonstrated that altered expression of kinesins is associated with development and progression of various human cancers. Kinesin family member 26B (KIF26B), a member of the kinesin superfamily proteins (KIFs), is essential for kidney development. However, the role of KIF26B during tumorigenesis and progression is limited. Here, we demonstrate that both KIF26B mRNA and protein are overexpression in breast cancer tissues by RT-qPCR and western blot. Immunohistochemistry revealed that KIF26B expression significantly correlated with clinicopathological factors, including tumor size (*P* = 0.011), grade (*P* = 0.017), lymph node status (*P* = 0.009) and ER status (*P* = 0.012). Moreover, the Kaplan-Meier analysis indicated that breast cancer patients with high KIF26B expression had a shorter survival than those with low KIF26B expression. In addition, multivariate analysis indicated that KIF26B is an independent prognostic for outcome in breast cancer (HR, 2.356; 95%CI, 1.268–4.378; *P* = 0.007). Collectively, our study demonstrated that KIF26B was overexpression in breast cancer and could be served as a potential prognostic marker.

## Introduction

Breast cancer is one of most common malignant tumors with 1st incidence and 2nd mortality among all malignant tumors in females [1]. Although improvements in early detection and treatment have decreased breast cancer mortality rates in recent years, prevention and therapy of breast cancer remain a major public health concern. Breast cancer is a heterogeneous disease with respect to molecular alteration, cellular composition, and clinical outcome [2]. Unfortunately, the incomplete understanding of its carcinogenic mechanisms leads to difficulties in selecting targeted treatment and contributes to a low survival rate for patients with breast cancer.

The kinesin superfamily proteins (KIFs) are a conserved class of microtubule-dependent molecular motor proteins and have an ATPase activity and motion characteristics, with more than 45 members have been identified in mammalian cells [3]. They provide power for a variety of transportations in cell cycle, are involved in membrane transport, mitosis and meiosis, mRNA and protein transport, signal transduction, and microtubule polymer dynamics [4]–[6]. Accumulating evidence demonstrates the importance of KIFs in the regulation of many physiological events, including brain function, developmental patterning, and even tumorigenesis [7]. Recently, more and more studies have been reported that the abnormal expression of kinesins plays a key role in the development or progression in a variety of human cancers. The abnormal kinesin expression will alter the equal distribution of genetic materials during the cell mitosis because of chromosome hypercondensation, aberrant spindle formation, anaphase bridges, defective cytokinesis, aneuploidy, and mitosis arrest. The loss or gain of the genetic materials will lead to numerous defects in the daughter cells, which could promote tumorigenesis and participate in regulation of tumor cell invasion and metastasis. Thus, Better understanding of kinesin protein functions may help develop novel molecular-targeted drugs for various human cancers [8]–[10].

KIF26B is classified to the Kinesin-11 family along with KIF26A, an unconventional that play a role in embryogenesis, specifically in the development of limbs, face, and somites [11]. The founding member of kinesin-11 family is Smy1, a member from Saccharomyces cerevisiae, which does not bind to microtubules, due to the deviance in both a catalytic pocket for ATP hydrolysis and the microtubule-binding sites [12]. Another member of Kinesin-11, KIF26A, does not have the conserved amino acid sequences that are required for motor activity but retains the microtubule-associating ability as well as other conserved KIFs and regulates GDNF-Ret signaling in enteric neuronal development [13]. Furthermore, KIF26B is essential for kidney development and its expression is restricted to the metanephric mesenchyme, and its transcription is regulated by a zinc finger transcriptional regulator Sall1 [14]. However, the role of KIF26B in tumorigenesis and progression is limited.

In this study, we examined KIF26B expression in breast cancer tissues, analyzed the relationship between KIF26B expression and clinicopathological factors, and determined the potential role of KIF26B in breast cancer prognostic prediction. Our data indicated that KIF26B was remarkably overexpression in breast cancer and could be served as a potential biomarker of prognosis.

## Materials and Methods

### Patients and specimens

All breast tissues, including 30 paired adjacent normal tissues and 200 primary breast cancer tissues were collected from 200 breast cancer patients. Surgery was performed from September 2000 to December 2005 at Hubei University of Medicine, Hubei, China. No patients received chemotherapy or radiotherapy prior to the operation. This study was approved by the Institutional Review Board of the Hubei University of Medicine and written consent was obtained from all participants.

### Western blot

50 µg of lysates was separated on 10% SDS-PAGE gels, transferred to polyvinylidene fluoride membranes (Millipore), blocked with 5% skimmed milk and then incubated with primary antibody specific for KIF26B (1∶2000, ab121992, Abcam) for 4°C overnight. The blots were then rinsed in Tris-buffered saline-Tween (TBST) and further incubated in peroxidase-conjugated anti-rabbit secondary antibody (1∶2500, sc-2004, Santa-Cruz biotechnology). Protein bands were visualized using the pico chemiluminescence (ECL) detection system (Pierce Biotech) and exposed to film. Mouse monoclonal antibody to β-actin (1∶8000, Sigma) was used as gel loading control.

### Immunohistochemistry

Immunohistochemistry (IHC) analysis for KIF26B using an ABC method. Briefly, after dewaxing and hydration, 4 µm sections from formalin fixed paraffin-embedded tissue were subjected to heat-induced epitope retrieval in 0.01 M citrate buffer (pH 6.0). Endogenous peroxidase activity was blocked in 3% hydrogen peroxide. Nonspecific binding was blocked by treatment with normal horse serum at room temperature. The slides were then incubated with primary polyclonal rabbit anti-KIF26B antibody (1∶300, Abcam, the same antibody used for Western blot) at room temperature. 3, 3′-diaminobenzidine (DAB) was used for color development, and hematoxylin was used for counterstaining. Negative control slides were processed without primary antibody and were included for each staining.

IHC was scored independently using a semi-quantitative scoring system as described below by two pathologists, who were both blinded to patients' clinicopathologic parameters and outcomes. Discordant scores were re-evaluated by the investigators and the consensus scores were used for further analyse. Using the H-score method [15], we multiplied the percentage score by the staining intensity score. Percentage of staining was categorized as “0” if there was no cytoplasm expression, “1” for up to 25% positive tumor cytoplasm, “2” for 26% to 50%, “3” for 51% to 75%, and “4” for 76% to 100%. Intensity was scored as “1”, “2”, and “3” for weak, moderate, and strong staining, respectively. Percentage (P) and intensity (I) of cytoplasm expression were multiplied to generate numerical score (S = P×I). The median H-score was chosen as cut-off point to separate “KIF26Bhigh” (H-score > median) from “KIF26B_low_” (H-score ≤ median) tumor samples.

### Quantitative real-time PCR

Total RNA from breast cancer tissues was isolated using the TRIzol® Reagent (Invitrogen) and re-suspended in sterile DEPC water. The amount of RNA was determined by spectrophotometry and sample integrity was monitored through visualization of ribosomal RNAs (28S and 18S) by electrophoresis. 5 µg of total RNA was used to convert to cDNA with RevertAid First Strand cDNA Synthesis Kit (Thermo Scientific). Real-time PCR analysis was performed using DyNAmo Flash Probe qPCR kit (Thermo Scientific) according to the manufacturer's instructions. Assays were performed with the ABI 7900 TaqMan system (Applied Biosystems) using following primers: 5′-AGGCCATGTGCTTCAATGCAAA-3′ (forward), 5′- ATCCAGCATCAGATACTGTTTGGT-3′ (reverse), and 5′-(FAM) CCAGCTCCTTCATCAGCCACTCG (TAMRA)-3′ (probe) (Sangon Biotech). The PCR conditions were carried out after incubation at 50°C for 2 min and pre-denaturing at 95°C for 3 min, followed by 40 cycles at 95°C for 30 sec and 62°C for 1 min. Experiments were performed in triplicate, for both the target and the housekeeping gene. A no-template control was included in each amplification reaction. The PCR efficiencies of the two genes were comparable (≥95%). Quantification of expression of the target gene in the samples was accomplished by measuring the fractional cycle number at which the amount of expression reached a fixed threshold (C_t_). The relative quantification was given by the C_t_ values, determined by triplicate reactions for all of the samples for both KIF26B and β-actin. The ΔC_t_ was determined by subtracting the β-actin C_t_ from the target gene C_t_. The relative expression level of KIF26B mRNA was determined as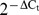.

### Statistical analysis

The Students' *t* test was used for comparison between groups. The χ^2^ test was performed to analyze the correlation between KIF26B expression and clinicopathological parameters. The Kaplan-Meier analysis (the log-rank test) was used for survival curves. Cox regression model with stepwise manner (forward, likelihood ratio) was utilized to perform a multivariate analysis. The level of significance was set to *P*<0.05.

## Results

### The mRNA and protein levels of KIF26B were upregulation in breast cancer tissues

**Figure 1:**
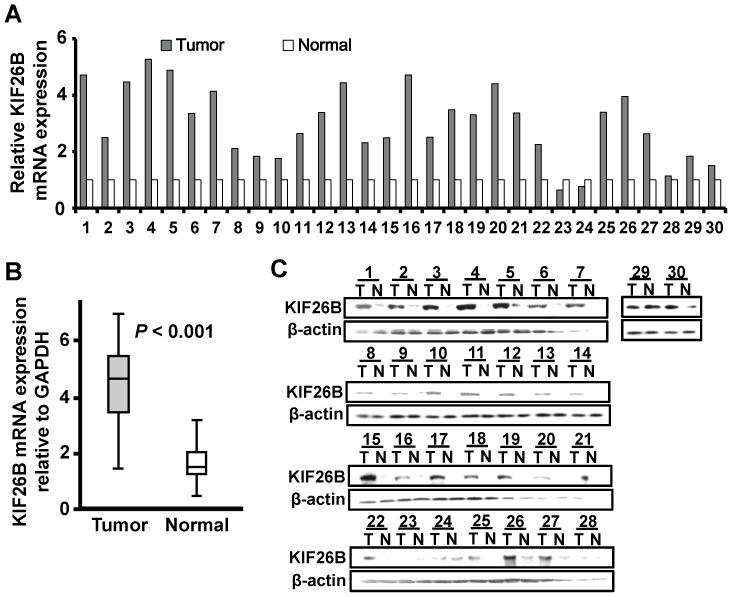
KIF26B mRNA and protein expression in breast cancer. **A,** KIF26B mRNA was upregulation in 27 of 30 breast cancer cases. The relative KIF26B mRNA expression was indicated by histogram (Tumor, primary breast cancer tissues; Normal, the paired adjacent normal breast tissues). **B,** The KIF26B mRNA levels was significantly upregulation in breast cancer as determined by matched paired Students' *t* test. **C,** KIF26B protein expression was upregulation in 26 of 30 breast cancer cases by western blot. β-actin was used as the loading control.

To examine expression of KIF26B in breast cancer tissues, we firstly detected the mRNA and protein levels in 30 paired primary breast cancer tissues and the corresponding adjacent normal tissues using RT-qPCR and western blot. In 27 of 30 cases, KIF26B mRNA was upregulated in primary breast cancer tissue, compared to the adjacent normal breast tissues (Fig. 1A and B, *P*<0.001). We found no differences between the 30 patients to the full pool of 200 patients (Fig. S1). Consistently, in 26 of 30 cases, the protein levels of KIF26B in primary breast cancer tissues were dramatically higher than those in the normal breast tissues by western blot (Fig. 1C). These results suggested that KIF26B is overexpression in breast cancer tissues.

### The relationship between KIF26B expression and clinicopathological factors

**Figure 2:**
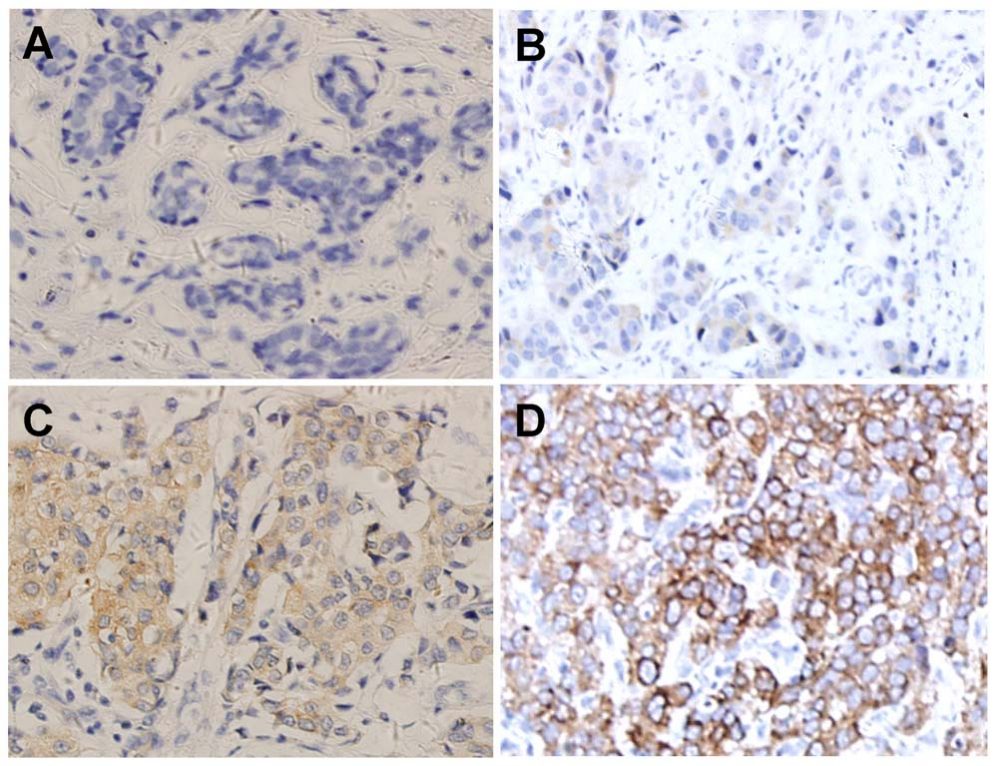
Immunohistochemical analysis of KIF26B in breast cancer. Normal breast contains rare KIF26B-positive cells **(A)**, whereas breast cancer tissues show weak **(B)**, moderate **(C)**, and strong **(D)** staining of KIF26B. Numerical scores: A = 0, B = 2, C = 6, D = 12.

Immunohistochemistry was performed to determine the KIF26B expression in 200 paraffin-embedded primary breast cancer tissues. Representative KIF26B immunostaining of normal breast and breast cancers is shown in Fig. 2. KIF26B expression was restricted to the cytoplasm with negligible nuclear staining. Tumor showed variable KIF26B expression: weak, moderate, and strong. High level of KIF26B expression (KIF26B_high_; score > median) was seen in 94 of 200 breast cancer. The relationship between KIF26B expression and clinicopathological factors was further analyzed. Significant correlations were found between KIF26B expression and four clinicopathological factors including tumor size (*P* = 0.011), lymph node status (*P* = 0.009), grade (*P* = 0.0017), and ER status (*P* = 0.012) (Table 1). There were no statistical connection between KIF26B expression and the other clinicopathological factors, such as age, menopausal status, clinical stage, PR, Her-2 and histology (*P*>0.05, Table 1).

**Table 1:** Relationship between clinicopathologic factors and KIF26B expression.

Variable	Cases	KIF26B_low_ (%)	KIF26B_high_ (%)	Chi-squae test	
				χ^2^	*P*
**Age (years)**				0.254	0.615
≥55	91	50 (54.9)	41 (45.1)		
<55	109	56 (51.4)	53 (48.6)		
**Menopausal status**				0.071	0.790
Pre-	98	51 (52.0)	47(48.0)		
Post-	102	55 (53.9)	47 (46.1)		
**Size (cm)**				6.533	0.011*
≤2	135	80 (59.3)	55(40.7)		
>2	65	26 (40.0)	39 (60.0)		
**Lymph node status**				6.899	0.009*
Negative	92	58 (63.0)	34 (37.0)		
Positive	108	48 (44.4)	60 (55.6)		
**Stage**				1.707	0.191
I-II	153	85 (55.6)	68 (44.4)		
III	47	21 (44.7)	26 (55.3)		
**Grade**				5.689	0.017*
I-II	159	92 (57.9)	69 (42.1)		
III	41	14 (34.1)	25 (65.9)		
**ER**				6.328	0.012*
Positive	122	56 (45.9)	66 (54.1)		
Negative	78	50 (64.1)	28 (35.9)		
**PR**				1.408	0.235
Positive	106	52 (49.1)	54 (50.9)		
Negative	94	54 (57.4)	40 (42.6)		
**Her-2**				0.000	0.995
Negative	134	71 (53.0)	63 (47.0)		
Positive	66	35 (53.0)	31 (47.0)		
**Histology**				0.002	0.963
Lobular	172	87	85		
Ductal	18	9	9		

* Statistical significant.

### Association of KIF26B expression with survival of patients with breast cancer

**Figure 3:**
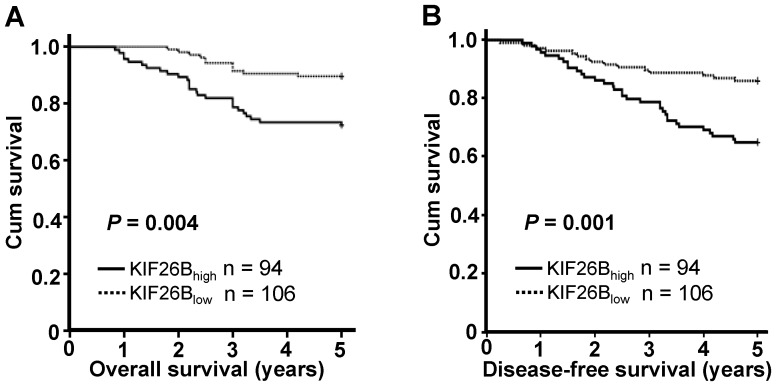
Kaplan-Meier analysis of cancer-specific survival. KIF26B protein levels showed prognostic role in overall survival **(A)** and disease-free survival **(B)**.

Kaplan-Meier analysis was used to evaluate the survival of patients with breast cancer. Patients with high KIF26B expression were likely to be with significantly shorter overall survival (*P* = 0.004, Fig. 3A) and disease-free survival (*P* = 0.001, Fig. 3B).

### Univariate and multivariate analyses of prognostic variables in breast cancer patients

We next evaluated the KIF26B expression and other clinicopathologic factors on prognosis of breast cancer, using univariate analyses. Results indicated that tumor size (HR: 1.933, 95% CI: 1.108–3.372, *P* = 0.02), Lymph node status (HR: 3.546, 95% CI: 1.815–6.928, *P*<0.001), clinical stage (HR: 2.940, 95% CI:1.680–5.144, *P*<0.001), grade (HR: 2.000, 95% CI: 1.104–3.624, *P* = 0.022) and KIF26B (HR: 1.919, 95% CI: 1.198–3.067, *P* = 0.006) as significant predictors of cancer-specific survival. ER, PR, and Her-2 did not significantly affect cancer-specific survival (Table 2).

**Table 2:** Univariate analysis of various variables.

Variable	Comparison	HR (95% CI)	*P*-Value
Tumor size	≤2 vs>2	1.933 (1.108–3.372)	0.020*
Lymph node status	N_0_ vs N_+_	3.546 (1.815–6.928)	<0.001*
Stage	I-II vs III	2.940 (1.680–5.144)	<0.001*
Grade	I-II vs III	2.000 (1.104–3.624)	0.022*
ER	ER+ vs ER-	1.576 (0.904–2.749)	0.109
PR	PR+ vs PR-	1.563 (0.894–2.733)	0.117
HER2	Her2+ vs Her2-	0.814 (0.460–1.441)	0.480
KIF26B	KIF26B_low_ vs KIF26B_high_	1.919 (1.198–3.067)	0.006*

* Statistical significant.

Furthermore, KIF26B expression and those clinicopathologic variables significant in univariate analysis (i.e., tumor size, lymph node status, clinical stage, grade, ER, and KIF26B) were further evaluated in multivariate analysis. Results indicated that lymph node status (HR: 2.548, 95% CI: 1.267–5.126, *P* = 0.009), clinical stage (HR: 2.273, 95% CI: 1.278–4.044, *P* = 0.005), and KIF26B (HR: 2.356, 95% CI: 1.268–4.378, *P* = 0.007) were also independent predictor for DFS of HCC patients (Table 3).

**Table 3:** Multivariate analysis of various variables.

Variable	Comparison	HR (95% CI)	*P*-Value
Tumor size	≤2 vs>2	1.983 (1.716–2.321)	0.159
Lymph node status	N_0_ vs N_+_	2.548 (1.267–5.126)	0.009*
Stage	I-II vs III	2.273 (1.278–4.044)	0.005*
Grade	I-II vs III	0.981 (0.884–1.507)	0.322
KIF26B	KIF26B_low_ vs KIF26B_high_	2.356 (1.268–4.378)	0.007*

* Statistical significant.

## Discussion

Kinesin was first discovered in 1985 [16]. To date, a total of 45 murine and human kinesin superfamily proteins (KIFs) have been identified and classified into 14 large families, termed kinesin 1 to 14 [3], [7], [17]. KIFs are a conserved class of microtubule-dependent molecular motor proteins with a wide range of cellular functions, including vesicle transport, mitotic spindle formation, chromosome segregation, midbody, formation, and cytokinesis completion [8], [18]. They are defined by the motor domain position at the N terminus (N-type), C terminus (C-type), and internal region (I-type) [19].

KIF26B, a mammalian kinesin classified as an N-type kinesin-11 family member, is essential for embryonic kidney development. KIF26B expression is restricted to the metanephric mesenchyme and plays an important role in the compact adhesion between mesenchymal cells adjacent to the ureteric buds, possibly by interacting with nonmuscle myosin [20]. To our knowledge, the study about KIF26B expression during tumorigenesis and progression is limited.

To date, a great number of studies have demonstrated that altered expression of kinesins is associated with development and progression of various human cancers [9], [21]–[23]. For example, several reports have been showed that the increased KIF14 expression is related to a variety of different cancers tumorigenesis and poor prognosis, including retinoblastoma, lung cancer, and even breast cancer [24]–[26]. Zhang et al. indicated that KIF18A promotes breast carcinogenesis and progression. Castillo and colleagues have demonstrated proof of principle of a role for KIF11 overexpression in driving tumorigenesis, by showing that transgenic mice overexpressing KIF11 are prone to the development of a variety of different tumor types [27]. Furthermore, more than 18 PhaseI or PhaseII clinical trials involving KIF11-targeting agents are ongoing or have been completed and these agents have been moderately successful when used as a monotherapy [9].

In the present study, we found that both KIF26B mRNA and protein were upregulated in primary breast cancer tissues. Furthermore, high expression of KIF26B in breast cancer was significantly associated with other malignant tumor characteristics, such as larger tumor size, higher histological grade and lymph node metastasis, indicating that KIF26B might be served as a hallmark of malignant tumors. In addition, we find that high KIF26B expression positively correlated with ER status, which suggested KIF26B may induce tumor proliferation. In the results of Kaplan-Meier survival analysis, we found that patients with low KIF26B expression had longer survival. This might be explained by the findings Sall1 transactivate the KIF26B expression [20], [28], and that the Sall1 protein was also shown to be highly expressed in tumors [29]. Furthermore, Cox regression analysis suggested KIF26B as an independent prognostic factor, indicating that KIF26B may be an important modulator involved in breast cancer development.

In conclusion, we showed that KIF26B is overexpression in breast cancer tissues. Moreover, our study provides the clinical evidence that KIF26B is independently prognostic for outcome in breast cancer. Independent validation of these clinical findings, examination of KIF26B expression in other kinds of cancers, and further investigation of the cell biology of KIF26B and its potential as a therapeutic target are clearly warranted.

## Supporting Information

Figure S1 **KIF26 mRNA expression in breast cancer tissues.** All, KIF26B mRNA expression in all of 200 patients. Part, KIF26B mRNA expression in 30 of 200 patients.
(TIFF)
