# Correction: High Expression of KIF26B in Breast Cancer Associates with Poor Prognosis

**DOI:** 10.1371/annotation/f7a306c2-3de5-4b59-9825-09e4cbc884bf

**Published:** 2013-06-10

**Authors:** Qun Wang, Zong-Bin Zhao, Geng Wang, Zhen Hui, Ming-Hua Wang, Jun-Feng Pan, Hong Zheng

There were errors in the two affiliations of the authors of the article.

The correct affiliations are, respectively:

1 Department of Endocrine and Vascular Surgery, Taihe Hospital, Hubei University of Medicine, Shiyan, P.R. China

2 Department of Preventive Medicine, Hubei University of Medecine, Shiyan, P. R. China 

