# Supplementary figures and images for: High Expression of KIF26B in Breast Cancer Associates with Poor Prognosis
Source: PLoS One. 2013 Apr 9;8(4):e61640. doi: 10.1371/journal.pone.0061640 (PMC3621833; doi:10.1371/journal.pone.0061640)

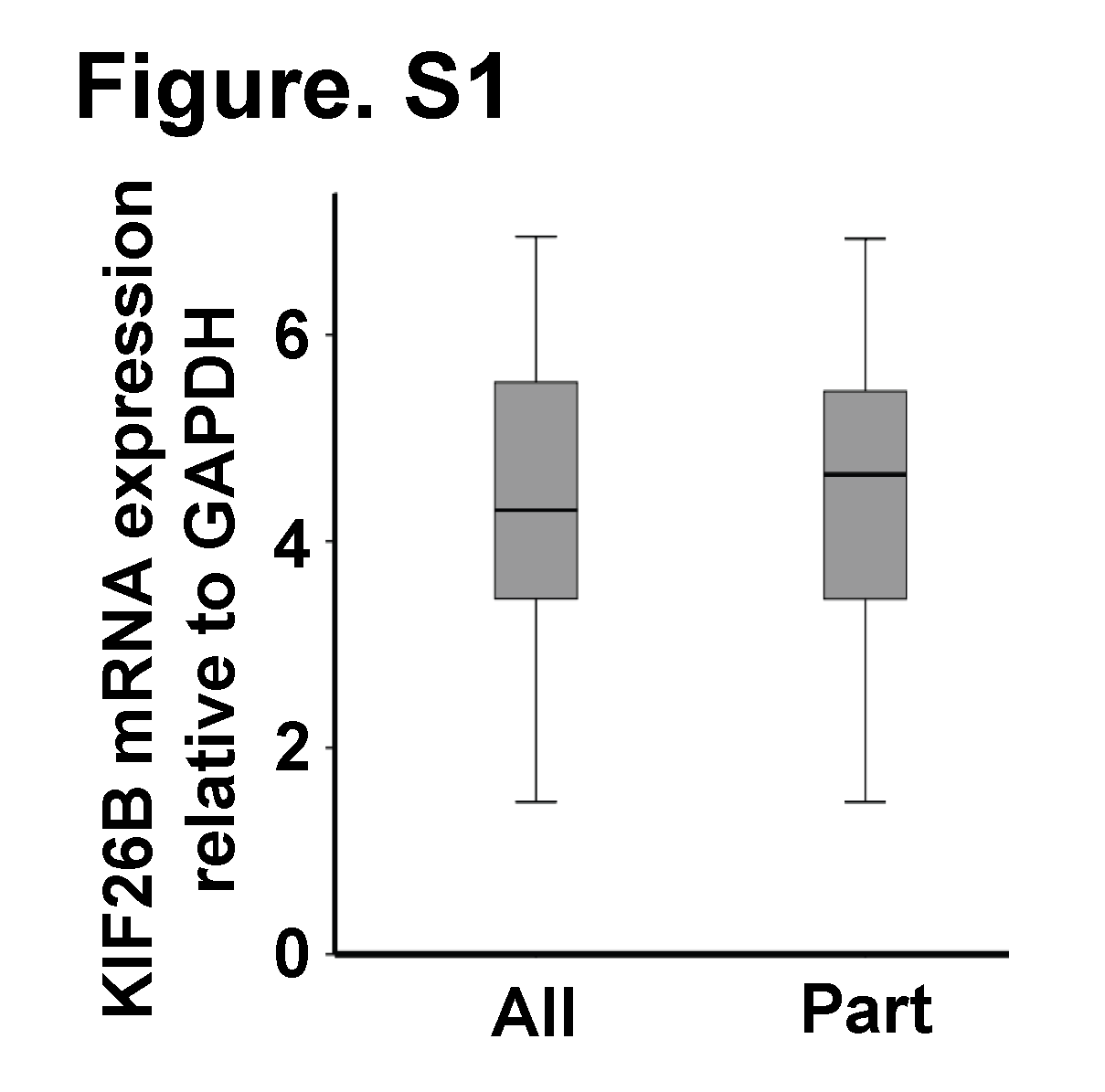

Supplement: Figure S1 — KIF26 mRNA expression in breast cancer tissues. All, KIF26B mRNA expression in all of 200 patients. Part, KIF26B mRNA expression in 30 of 200 patients. (TIFF) [file pone.0061640.s001.tiff]
